# Cluster randomised controlled trial of a theory-based multiple behaviour change intervention aimed at healthcare professionals to improve their management of type 2 diabetes in primary care

**DOI:** 10.1186/s13012-018-0754-5

**Published:** 2018-05-02

**Authors:** Justin Presseau, Joan Mackintosh, Gillian Hawthorne, Jill J. Francis, Marie Johnston, Jeremy M. Grimshaw, Nick Steen, Tom Coulthard, Heather Brown, Eileen Kaner, Marko Elovainio, Falko F. Sniehotta

**Affiliations:** 1 Ottawa Hospital Research Institute, The Ottawa Hospital-General Campus, 501 Smyth Road, Ottawa, ON K1H 8L6 Canada; 2 School of Epidemiology and Public Health, University of Ottawa, Ottawa, Canada; 3 Institute of Health and Society, Newcastle University, Newcastle upon Tyne, UK; 4 School of Health Sciences, City, University of London, London, UK; 5 Institute of Applied Health Sciences, University of Aberdeen, Aberdeen, UK; 6 Department of Medicine, University of Ottawa, Ottawa, Canada; 7 Benfield Park Medical Group, Newcastle upon Tyne, UK; 8 Department of Psychology and Logopedics, University of Helsinki, Helsinki, Finland; 9 Fuse, The UK CRC Centre of Excellence for Translational Research in Public Health, Newcastle upon Tyne, UK

**Keywords:** Diabetes, Health care professional, Behaviour change, Primary care, Cluster randomized trial, Blood pressure, HbA_1c_, Lifestyle advice, Foot examination, Theory, Multiple behaviours

## Abstract

**Background:**

National diabetes audits in the UK show room for improvement in the quality of care delivered to people with type 2 diabetes in primary care. Systematic reviews of quality improvement interventions show that such approaches can be effective but there is wide variability between trials and little understanding concerning what explains this variability. A national cohort study of primary care across 99 UK practices identified modifiable predictors of healthcare professionals’ prescribing, advising and foot examination.

Our objective was to evaluate the effectiveness of an implementation intervention to improve six guideline-recommended health professional behaviours in managing type 2 diabetes in primary care: prescribing for blood pressure and glycaemic control, providing physical activity and nutrition advice and providing updated diabetes education and foot examination.

**Methods:**

Two-armed cluster randomised trial involving 44 general practices. Primary outcomes (at 12 months follow-up): from electronic medical records, the proportion of patients receiving additional prescriptions for blood pressure and insulin initiation for glycaemic control and having a foot examination; and from a patient survey of a random sample of 100 patients per practice, reported receipt of updated diabetes education and physical activity and nutrition advice.

**Results:**

The implementation intervention did not lead to statistically significant improvement on any of the six clinical behaviours. 1,138,105 prescriptions were assessed. Intervention (29% to 37% patients) and control arms (31% to 35%) increased insulin initiation relative to baseline but were not statistically significantly different at follow-up (IRR 1.18, 95%CI 0.95–1.48). Intervention (45% to 53%) and control practices (45% to 50%) increased blood pressure prescription from baseline to follow-up but were not statistically significantly different at follow-up (IRR 1.05, 95%CI 0.96 to 1.16). Intervention (75 to 78%) and control practices (74 to 79%) increased foot examination relative to baseline; control practices increased statistically significantly more (OR 0.84, 95%CI 0.75–0.94). Fewer patients in intervention (33%) than control practices (40%) reported receiving updated diabetes education (OR = 0.74, 95%CI 0.57–0.97). No statistically significant differences were observed in patient reports of having had a discussion about nutrition (intervention = 73%; control = 72%; OR = 0.98, 95%CI 0.59–1.64) or physical activity (intervention = 57%; control = 62%; OR = 0.79, 95%CI 0.56–1.11). Development and delivery of the intervention cost £1191 per practice.

**Conclusions:**

There was no measurable benefit to practices’ participation in this intervention. Despite widespread use of outreach interventions worldwide, there is a need to better understand which techniques at which intensity are optimally suited to address the multiple clinical behaviours involved in improving care for type 2 diabetes.

**Trial registration:**

ISRCTN, ISRCTN66498413. Registered April 4, 2013

**Electronic supplementary material:**

The online version of this article (10.1186/s13012-018-0754-5) contains supplementary material, which is available to authorized users.

## Background

The estimated global prevalence of diabetes stands at 8.5% of adults, with resulting healthcare expenditures required to support patients and populations in managing avoidable diabetes-related morbidity [1]. Type 2 diabetes is managed in primary care in many jurisdictions, including the North East of England, which has a 6% estimated prevalence [2]. There are widely agreed clinical guidelines highlighting best practice and target-based incentive schemes promoting high-quality healthcare [3, 4]. While some data from the Quality and Outcomes Framework (QOF, the UK’s target-based incentive program) suggest diabetes care is improving, the most demanding indicators show room for improvement (e.g. blood pressure and glycaemic control [5]). National diabetes audits also suggest that people with diabetes are not receiving optimal healthcare: under 50% receive nine key aspects of care, with variation nationwide [6]. There remains an imperative to support healthcare professionals in improving quality of care to support patients in achieving optimal health outcomes.

We previously conducted a UK-wide study of 99 general practices in the UK MRC GP Research Network about their provision of care for type 2 diabetes, identifying opportunities for improvement (iQuaD study [7]). Patient records were audited, and those with type 2 diabetes with a guideline-defined clinical indication for increased medication for glycaemic control and hypertension were assessed for medication alteration in the previous 25 months. Only 39.5% (1595/4038; 81 practices) and 58.9% (624/1059; 81 practices) of eligible patients were prescribed additional therapy for hypertension and glycaemic control, respectively. Of 29,362 patient records assessed across 86 practices, 77.1% had a foot examination. In a patient survey assessing recall of care received over the past year involving 3591 patients with type 2 diabetes, 94% reported attending their practice in the last year [8]. Only 51% of those with a BMI over 30 reported receiving weight management advice, 68% reported receiving self-management advice, and 73% reported receiving information about diabetes [7, 8].

Many factors contribute to variations in care (e.g. patient physiology, treatment response and preferences of patients and their families). However, it is also a function of barriers and enablers to healthcare professional behaviour that, if addressed, could optimize care. iQuaD identified modifiable determinants of clinical behaviours that predicted subsequent behaviour [7–12]. Practice structures of care and views about organization and team function were not strong predictors of care. However, factors including motivation, self-efficacy, action and coping planning and habit were consistently predictive of the six clinical behaviours, providing a basis for intervention development.

Reviews of trials of quality improvement interventions for diabetes care show they can be effective [13]. However, there is wide variability in effects and little understanding of what explains such variability or the theoretical and conceptual rationale for selecting intervention strategies [13–15] in ways that add to those already in place (e.g. QOF).

Many quality improvement interventions focus on a single healthcare professional behaviour. However, clinicians advise, assess, test, review, counsel, measure, prescribe and refer all in time-limited consultations [16, 17]. We developed a multiple behaviour change intervention aimed at primary care health professionals to improve their management of type 2 diabetes and evaluated it in a cluster randomized controlled trial to minimize contamination. We hypothesized that more patients with type 2 diabetes in practices receiving the intervention would receive guideline-consistent prescribing, advice and foot examination than those in control practices.

## Methods

### Trial design

As described in the trial protocol [18], we conducted a two-armed cluster randomized controlled trial. The primary care practice was the unit of randomization. The intervention was delivered to the primary care team, aiming to improve care provided by primary care health professionals.

### Sample size

The trial was powered to detect differences between intervention and control practices in the six clinical behaviours, anticipating an intra-cluster correlation coefficient of 0.06. Sample size estimation showed that 20 patients from 40 practices were required to detect a 15% absolute improvement in any of our six clinical behaviours with 80% power at 5% significance levels. To protect against cluster-level attrition, we aimed to recruit 44 practices, providing 83.9% power to detect a 15% improvement if all practices were retained.

### Participants

#### General practice (clusters) recruitment and data collection

General practices in North-East England and the general medical practitioners (GPs), nurses and healthcare assistants within them involved in providing care for type 2 diabetes participated. We excluded one practice involved in feasibility piloting. We aimed to recruit 44 practices across North-East England. Initial recruitment and contact was via research facilitators. The trial team also presented at a GP research forum. Interested practices contacted us, and we obtained written practice-level consent. We conducted phone interviews with practice contacts to assess organizational structures and identify staff involved in diabetes care. Practices randomized to the intervention were reimbursed for staff time to prepare for and attend the intervention session at an hourly staff rate.

#### Healthcare professional recruitment and data collection

Consent was sought from the cluster level and the general practice staff level prior to randomization. An information and consent sheet was mailed to identified GPs, nurses and healthcare assistants in each practice. We did not involve community nurses working with multiple practices to reduce contamination risk between intervention and control practices. As part of a planned process evaluation, a baseline and 12-month follow-up questionnaire was posted to GPs, nurses and healthcare assistants in all practices.

#### Trial patients recruitment and data collection

We included patients 18 and older with type 2 diabetes registered in each practice. Patients were identified in computerized medical records using diabetes-identifying codes. We excluded patients under 18, with type 1 diabetes, or for whom inclusion was clinically inappropriate as judged by each primary care team.

### Intervention description

Intervention development was consistent with the UK MRC framework for complex interventions [19] and informed by our national study of diabetes care (iQuaD [7]). We used behaviour change theory results from iQuaD to identify factors to target and included behaviour change techniques (BCTs) specified within these theories (Social Cognitive Theory, Health Action Process Approach, Dual Process Model, theory-based approaches to multiple goal pursuit) [16, 20]. The overarching strategy involved outreach visits [21] that moved beyond a knowledge and education-based approach that is more common of such interventions and instead emphasized healthcare professional behaviour change and addressed  clinical inertia. In brief, the intervention consisted of a content expert (nurse or MD) and a behaviour change expert delivering the intervention at each of 22 individual practices. The intervention allowed practice team members to dedicate 90 min together to consider each of the targeted clinical behaviors and select the behaviors consistent with their current role in the practice. They considered discrepancies between personal estimates of current performance and intended levels and identified barriers and problem solved by developing if-then plans to circumvent identified barriers. They were provided with materials developed with patients with diabetes to pre-identify barriers and solutions to assist in providing physical activity and healthy eating support [22–24]. We produced short videos using trained actors presenting practice-based examples of interactions between patients and clinicians (available upon request), common barriers to initiating insulin and providing physical activity advice and possible solutions for successfully managing barriers (see online Additional files 1, 2 and 3 for detailed intervention description).

#### Feasibility

We conducted two feasibility tests in primary care settings. The first was delivered to medical students to assess pace and timing and identify misunderstandings. The second involved two GPs, two nurses and a healthcare assistant at one practice (excluded from trial recruitment).

### Control practices

Control practices supported outcome measure collection (patient survey at 12 months follow-up and access to anonymised patient records at 12 months follow-up); no intervention was provided. Clinicians in control practices completed questionnaires at baseline and 12-month follow-up (planned secondary analysis). We sent materials to control practices at the end of the trial.

### Primary outcomes: six clinical behaviours

Primary outcomes were pre-specified in the protocol [18] and consistent with the UK NICE diabetes quality standards [4] (detailed outcome description in online Additional file 4). Outcome data were collected 12 months after intervention delivery. For the two *prescribing* behaviours and *foot examination*, outcome data were extracted from anonymised patient electronic records for all patients meeting inclusion criteria in each practice for 12 months before and 12 months after the intervention period. While data on *prescribing* and *foot examination* were available in routinely collected data in patient electronic records, providing advice and updated diabetes education was known to not consistently be recorded in medical records [7]. Outcome data on providing advice on *nutrition* and *physical activity* and providing updated diabetes *education* were therefore collected from anonymized postal survey responses from a random sample of 100 patients with type 2 diabetes in each practice at 12 months follow-up; no pre-intervention data were collected for outcomes assessed with patient surveys. Patients reported whether they had received physical activity advice, nutrition advice and updated diabetes education in the preceding 12 months. We developed patient surveys based on our previous study [7] and pilot-tested the surveys with our local Diabetes UK patient group. Practices managed processes relating to patient identification and questionnaire mailing to maintain patient anonymity to the trial team. One reminder was sent. Completed questionnaires were returned to the trial team with practice ID as the only identifier.

### Randomization, allocation and concealment

Practices were randomized 1:1 to intervention or control by the trial statistician using computer-generated random permuted blocks with practice roster (list) size as the blocking factor. The study research associate enrolled the clusters (practices). Baseline data collection (interviews and questionnaires) was completed before practices were made aware of their allocation. The study research associate assigned a unique study ID to each practice, and then, the trial statistician undertook the randomization of these IDs. The statistician and all trial team members except the trial research associate remained blind to practice allocation until the trial statistician completed the outcome analyses.

For *prescribing* behaviours and *foot examination*, an independent outcome assessor extracted data on healthcare professional behaviours using computerized medical records and remained blind to allocation. Clinical behaviour outcomes assessed using patient surveys of care received involved administrative staff in each practice. This data collection occurred 12 months after the intervention was delivered, at which point it is likely that administrative staff were aware of the allocation of their practice. Patients were not aware of the allocation.

### Analysis

We treated all six outcomes equivalently and hypothesized that each would show improvement in the intervention arm compared with control. To protect against type 1 error, our protocol pre-specified that the intervention should show a benefit on at least two behaviours to be regarded effective. We used different modelling approaches depending on characteristics of the data.

#### Prescribing for blood pressure and prescribing insulin for glycaemic control

The number of patients prescribed was analyzed using separate Poisson regression models. The number of patients who met target for triggering insulin initiation and additional prescribing for blood pressure post-intervention was included as an exposure variable. Baseline performance (proportion of patients meeting target threshold in the 6 months leading up to the intervention) and practice size were included as covariates. The impact of the intervention (randomization group) was included as a fixed effect.

#### Examining feet

The proportion of patients having their feet examined was analysed using a mixed effects logistic regression model. Fixed effects included the data time period (before or after the intervention) and the randomization group. Random effects took into account that patients were nested in practices. Additionally, an interaction between time period and group was included to test the effectiveness of the intervention.

#### Providing advice and education

We fitted a multilevel logistic model with patients nested within practices for each of the three clinical behaviour outcomes assessed by patient questionnaire at 12 months. As pre-specified, all analyses adjusted for any difference between practices in terms of list (roster) size.

### Cost analysis

We compared intervention and control practices on total prescription costs for diabetes and hypertension medication, costs of diabetes-related health service use, and intervention-associated costs. Prescription costs were collected for each practice for 12 months pre- and 12 months post-intervention. We extracted data on medication name, size, dose and prescription date for all eligible patients included in the trial analyses of the primary outcomes. Diabetes and blood pressure medication costs were extracted from anonymised patient medical records in all 44 practices, consisting of 1,138,105 recorded prescriptions between 18 August 2012 and 28 February 2015. The total cost of prescriptions for diabetes and hypertension was calculated using the prescription length and cost of the medication. Medication costs were taken from the British National Formulary archive [25] and were deflated to 2012–2013 prices and assumed the same rate of inflation for 2015 [26]. Service use and referral costs were calculated from patient questionnaire responses using costs data from the Unit Costs of Healthcare [27] and NHS reference costs [28] and deflated to 2012–2013 prices. Post-intervention service use costs were calculated as the post-intervention equivalized total cost per patient for each practice. This provides a cost per patient for each practice, accounting for practice size. Patients in each practice were allocated a mean cost for their practice. The intervention costs were provided for all intervention practices.

## Results

### Recruitment

**Fig. 1:**
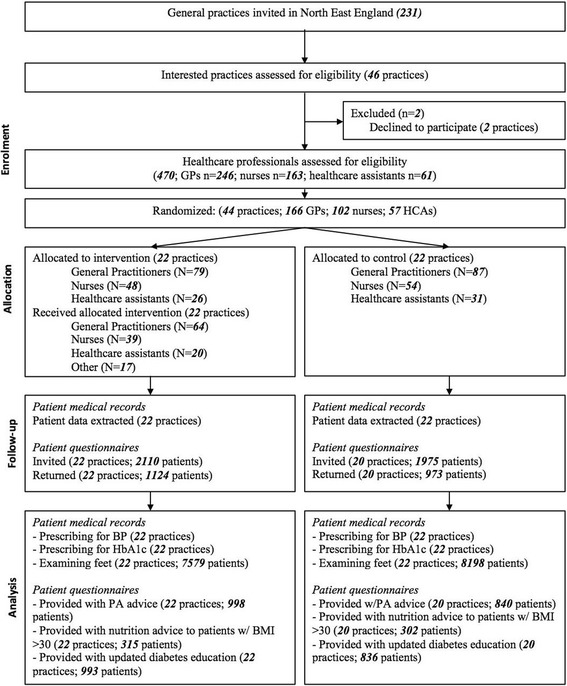
IDEA trial—CONSORT flow diagram

We recruited 44 practices from March 2013 to November 2013 and randomized 22 to the intervention (153 GPs, nurses, and HCAs) and 22 (172 GPs, nurses, and HCAs) to the control. Table 1 describes characteristics of the practices recruited, and Fig. 1 presents the CONSORT flow diagram. The intervention was delivered to each of the 22 practices in the intervention arm. One hundred twenty-three (80.4%) GPs, nurses and healthcare assistants were present at the intervention sessions. Seventeen other members of the practices also attended interventions sessions, including medical students on placement and administrative staff.

**Table 1:** Baseline characteristics of general practices randomized to receive the intervention or not. Figures are means across practices (standard deviations)

	Intervention (*n* = 22)	Control (*n* = 22)
Number of urban practices	11	14
Number of semi-rural practices	5	5
Number of rural practices	6	3
Number of dispensing practices	7	5
Number of training practices	16	18
Mean list (roster) size at baseline (SD)	7031 (3698)	7113 (4189)
% of patients > 65 years old (SD)	20.50% (5.8)	18.22% (7.1)
% of patients with T2 diabetes (mean, SD)	4.73 (0.77)	4.77 (1.11)
Number of GPs (SD)	3.59 (2.22)	3.95 (2.98)
Number of nurses (SD)	1.92 (1.00)	1.89 (1.03)

### Outcome data collection

#### Anonymised patient records

We extracted anonymised prescribing data from 1,138,105 prescriptions across 44 practices between December 2014 and March 2015.

#### Patient survey

Four thousand eight-five (2110 intervention; 1975 control) patients were sent questionnaires from 42 practices. Two practices in the control condition were not able to send questionnaires in time for the outcome analysis; these practices were not included in the analyses based on patient questionnaire responses but are included in the analyses involving prescribing and foot examination. Two thousand ninety-seven (51.3%) patients returned a completed questionnaire (1124 intervention [53.3%]; 973 control [49.3%]).

### Primary trial outcome results

No statistically significant benefit of the intervention was detected on any of the six healthcare professional behaviours targeted by the intervention (see Tables 2 and 3).

**Table 2:** Results of prescribing for HbA_1c_, prescribing for blood pressure and examining feet based on patient electronic records in intervention (*n* = 22) and control (*n* = 22) practices

	Baseline		Follow-up (12 months)				
Outcome (clinician behaviour)	Intervention	Control	Intervention	Control	Effect size (95%CI)	*p*	ICC
Prescribed additional therapy for glycemic control^1^	29%	31%	37%	35%	IRR 1.18 (0.95 to 1.48)	0.13	–
	(95%CI 21–36%)	(95%CI 22–41%)	(95%CI 31–43%)	(95%CI 29–41%)			
Prescribed additional therapy for blood pressure^2^	45%	45%	53%	50%	IRR 1.05 (0.96 to 1.16)	0.29	–
	(95%CI 37–52%)	(95%CI 42–47%)	(95%CI 47–59%)	(95%CI 46–53%)			
Examined circulation and sensation in feet^3^	75%	74%	78%	79%	OR 0.84 (0.75 to 0.94)	< 0.01	0.03

Note: Percentages based on uncorrected means. Effect sizes adjusted for baseline and practice size. ICC not calculated for prescribing as unit of analysis was the practice level

*IRR* incidence rate ratio, *OR* odds ratio, *ICC* intracluster correlation

^1^Numerator: number of patients prescribed insulin for the first time. Denominator: Number of patients with type 2 diabetes in each practice with an HbA_1c_ measure exceeding 58 mmol/mol (7.5%) at least once in the 12 months post-intervention who were also on three or more oral hypoglycemic drugs

^2^Numerator: number of patients prescribed an additional antihypertensive drug or had tablet size changed. Denominator: patients with type 2 diabetes in each practice with at least one blood pressure measure above 140/80 mmHg, excluding those with a subsequent follow-up measure below blood pressure threshold

^3^Numerator: patients with recorded examination of sensation and circulation in each foot. Denominator: all patients meeting inclusion criteria

**Table 3:** Results of providing nutrition advice, providing on-going diabetes education and providing physical activity advice, based on patient report at 12 months

	Mean at 12 months follow-up				
Outcome	Intervention (*n* = 22)	Control (*n* = 20)	OR (95%CI)	*p*	ICC
Provide personalized nutrition advice to those with BMI > 30	73% (*n* = 315)	72% (*n* = 302)	0.98 (0.59 to 1.64)	0.95	0.11
Provide on-going diabetes education	33% (*n* = 326)	40% (*n* = 330)	0.74 (0.57 to 0.97)	0.03	0.03
Provide personalized physical activity advice	57% (*n* = 568)	62% (*n* = 522)	0.79 (0.56 to 1.11)	0.17	0.06

Reported OR adjusted for practice size

*OR* odds ratio, *ICC* intracluster correlation

#### Prescribing and foot examination (patient medical records)

Both intervention (29 to 37% of eligible patients prescribed) and control arms (31 to 35%) showed increased insulin initiation in eligible patients relative to baseline but were not significantly different from each other at follow-up (incidence rate ratio 1.18, 95% CI 0.95 to 1.48, *p* = 0.13). Similarly, both intervention (45 to 53%) and control practices (45 to 50%) increased prescription for blood pressure but were not significantly different from each other at 12 months follow-up (IRR 1.05, 95% CI 0.96 to 1.16, *p* = 0.29). Both intervention (75 to 78%) and control practices (74 to 79%) increased foot examination relative to baseline levels, with control practices increasing statistically significantly more (OR 0.84, 95% CI 0.75 to 0.94, *p* < 0.01).

#### Patient-reported provision of lifestyle advice and diabetes education (at 12 months)

A statistically significantly lower percentage of patients in intervention practices (33%) than control practices (40%) reported receiving updated diabetes advice (OR = 0.74, 95% CI 0.57 to 0.97, *p* = 0.03) at 12-months follow-up. Intervention and control practices did not differ in patient reports of having had a discussion about nutrition (intervention = 73%; control = 72%; OR = 0.98, 95% CI 0.59 to 1.64, *p* = 0.95) or physical activity (intervention = 57%; control = 62%; OR = 0.79, 95% CI 0.56 to 1.11, *p* = 0.17).

### Cost analysis

Mean pre-intervention per patient prescription costs for injectable medication to manage glycaemic control was £6531 (95%CI £6237 to £6824) in intervention and £7205 (95%CI £6911 to £7499) in control practices. Costs fell to £6081 (95%CI £5806 to £6357) in intervention practices and £6570 (95%CI £6313 to £6827) in control practices post-intervention. Post-intervention log-transformed costs per patient did not differ between groups (*p* = 0.25). Blood pressure prescription costs also fell post-intervention, from £96 (95%CI £92 to £99) and £89 (95%CI £83 to £94) in intervention and control to £92 (£95%CI £89 to £96) and £84 (95%CI £78 to £88) respectively. Patient-reported service usage costs are based on responses from 2011 people with diabetes from 20 intervention (*n* = 1039) and 20 control (*n* = 972) practices; two control practices did not return patient questionnaires, and thus, two intervention practices of similar size were randomly removed from the data. Service use costs were significantly higher (*p* < .001) in intervention practices (mean = £24.46 per patient, 95%CI £23.90–£25.03) compared to control practices post-intervention (mean = £21.61 per patient, 95%CI £20.92 to £22.31), although the absolute difference of £2.85 in costs per patient is relatively small. The intervention cost £1191 per practice for the research team to develop and deliver (see online Additional file 5).

## Discussion

### Statement of principal findings

Primary care practices receiving the intervention did not demonstrate significantly better performance on any targeted guideline-recommended clinical behaviours relative to control practices . For prescribing behaviours and foot examination, intervention and control arms improved at 12 months relative to baseline, but this cannot be attributed to the intervention. Foot examination rates were statistically significantly higher at follow-up in control (79%) compared to intervention practices (78%) when controlling for baseline, as were patient-reported levels of updated diabetes education (40% of patients in control practices and 33% in intervention practices). Prescription costs were not significantly different in patients in intervention practices relative to control practices (see Additional file 6 for CONSORT checklist).

### Strengths and weaknesses of the study

Strengths include strong recruitment and retention of practices and healthcare professionals, outcome data collection from routinely collected data in electronic records and patient-reported outcomes and a robust intervention development process informed by substantial prior empirical research and behaviour change theory, interdisciplinary content development, intervention delivered by content and behaviour change experts and feasibility testing.

Patient-reported outcomes have some limitations. First, recall-based measures are inherently limited by recall bias, though bias will be balanced across arms. Second, two control practices did not send patient questionnaires. Third, to maintain anonymity, practices were responsible for selecting a random sample of patients to whom to send questionnaires, potentially introducing selection bias. Fourth, the 51% response rate for the patient survey may suggest response bias; responding patients may have presented their healthcare providers more favourably, but this would be balanced across arms. A lower proportion of control patients (49%) returned a questionnaire than intervention (53%), though there is little reason to suspect their awareness of the intervention. Finally, as we only had post-intervention data for patient-reported outcomes, these analyses do not account for baseline.

The main behaviour change technique targeting self-efficacy was ‘demonstration of the behaviour’, involving a video presenting a primary care clinician successfully performing the behaviour with a patient despite barriers. Due to time constraints, this was developed and presented only for prescribing for HbA_1c_ and providing physical activity advice. The assumption that effects would generalize to the other behaviours was likely insufficient. Video coverage across all behaviours and more active techniques for building self-efficacy were planned, but feasibility testing showed it not to be feasible within 90 min. Such limitations exemplify trade-offs between desirable and logistically possible intervention content in this setting.

### Strengths and weaknesses in relation to other studies

Primary care-based diabetes management requires multiple clinical behaviours to optimize health outcomes. Delivering interventions which bundle behaviours reflects the realities of primary care [16]. Indeed, trials of quality improvement interventions often target multiple behaviours, such as multiple drug prescriptions and processes of care targets. As some such interventions are effective and others not, it does not seem that the number of target behaviours is necessarily an explanation for the lack of effect. Interventions in other settings suggest that targeting two behaviours concurrently is as effective as targeting them in sequence [17, 29]. However, there may be an inverse U-shaped curve where targeting an increasing number of behaviours may be beneficial only to a point [30]. Targeting multiple behaviours concurrently is practical and ensures optimal use of time. Future interventions should investigate whether targeting behaviours in parallel or in sequence is equally effective.

### Mechanisms and implications

An intervention may fail to be effective for a range of reasons [31]:

(a) Theory selection - The theoretical foundations may be inaccurate or inappropriate: This intervention was based on a foundation of research involving testing organizational, team and behaviour change theory, the latter providing consistent evidence of factors accounting for variability in healthcare provider behaviour, which served as the precursor to developing the current intervention. Future research could investigate alternative theories which may identify supplementary approaches that build on the present intervention’s theoretical foundation. Rather than atheoretical interventions, which have a long history of scattered non-cumulative evidence, theory provides coherence and the capacity for developing a stronger, cumulative evidence-base (cf [32]).

(b) Behaviour change technique selection and operationalization - techniques may be ineffective or may have been delivered at too low intensity [33]: The techniques in this intervention have a basis in empirical literature. However, it seems likely that delivery intensity (one 90-min session covering six clinical behaviours) may have been constrained by feasibility considerations and may have been insufficient. Interventions delivered with too low intensity may incorrectly lead to the conclusion that the intervention is ineffective [34];

(c) Environmental context - the context of delivery of the intervention and care setting may conflict with proposed change: Our problem solving-based intervention allowed for inherent tailoring to the individual practice settings. However, the intervention did not alter the choice architecture [35] nor did the intervention change specific environmental cues that may be supporting pre-existing practices;

(d) Control group - may inadvertently receive an equally effective intervention (including by contamination), and other co-occurring changes may mask intervention effects [36]: The intervention was delivered in a region active in diabetes quality improvement and focused on clinical behaviours targeted by wider-reaching interventions (e.g. QOF). This ‘rising tide’ effect [37] was evidenced in the three behaviours for which we have pre- and post-data, where there was evidence that both intervention and control practices improved relative to baseline;

(e) Recipients targeted - may not be those delivering sub-optimum care: Eighty percent of invited GPs, nurses, and healthcare assistants in the intervention condition attended the intervention;

(f) Fidelity of delivery - the intervention may be poorly delivered or delivered with low fidelity to protocol: We assessed fidelity of delivery by coding transcribed audio recordings of all intervention sessions and found that the intervention was delivered with high fidelity;

(g) Evaluation time point - follow-up period may be too long or too short to detect an effect: Given annual review GP visits, a 12-month follow-up was the most appropriate length of time. While possible that intervention effects may have been seen earlier and tapered off, this intervention was not of sufficient intensity for effects to last to 12 months. Our planned theory-based process evaluation will allow us to explore some of these possible explanations.

### Unanswered questions and future research

Designing implementation interventions often involves trade-offs between desirable (sometimes time consuming) activities and the practical logistics of delivery in routine general practice. Our piloting indicated that 90 min was the maximum acceptable time that practices could fit into schedules, and thus, the intervention was developed within these constraints. While tempting, a longer intervention or more sessions did not fit within general practice availabilities and may have undermined recruitment and retention. It is possible that the acceptable delivery duration constrained the intervention to an intensity inadequate to produce the level of behaviour change required to show effectiveness [34].

Trials of interventions that do not show intervention-attributable benefit can nevertheless contribute to the broader evidence base. Process evaluations examining fidelity to intervention content and activation of hypothesized mechanisms of action can shed light into an otherwise black box of effects (or lack thereof) [31, 38–40]. Such process evaluations are planned as future research.

## Conclusions

This trial evaluated an intervention aiming to improve six guideline recommended behaviours for type 2 diabetes delivered using outreach visits to primary care teams that went well-beyond knowledge-based educational outreach and showed no evidence of its effectiveness. As healthcare organizations continue to develop, deliver and seek support to optimize care using outreach visits, there is a need to better understand which techniques are most effective, at what level of intensity within available practical constraints, for improving the multiple clinical behaviours involved in managing type 2 diabetes. Drawing on theory and evidence provides a basis for not only one-off evaluations, but also for developing a cumulative evidence base of how to optimize outreach-based intervention content. A commitment to robust and iterative effectiveness evaluation of techniques tested in outreach-based interventions can help to further ensure that an evidence base can accrue to support stakeholder decision-making.

## Additional files

Additional file 1: TIDieR-based intervention description for the IDEA trial. (DOCX 33 kb)

Additional file 2: Labels and descriptions of behaviour change techniques delivered during the IDEA intervention. (DOCX 30 kb)

Additional file 3: TIDieR-based description of training provided to facilitators in the IDEA trial. (DOCX 30 kb)

Additional file 4: Main outcome definitions and operationalizations. (DOCX 32 kb)

Additional file 5: Cost of delivering the IDEA intervention in primary care (*n* = 22). (DOCX 34 kb)

Additional file 6: CONSORT checklist. (DOCX 42 kb)

## References

[1] Roglic G, World Health Organization (2016). Global report on diabetes.

[2] Health and Social Care Information Centre. Quality and Outcomes Framework–Prevalence, Achievements and Exceptions Report England, 2014-15 [Internet]. Health and Social Care Information Centre; 2015 [cited 2016 Sep 7]. Report No.: 1.1. Available from: https://digital.nhs.uk/data-and-information/publications/statistical/quality-and-outcomes-framework-achievement-prevalence-and-exceptions-data/quality-and-outcomes-framework-qof-2014-15.

[3] NICE. Type 2 diabetes in adults: management. National Institute for Health and Care Excellence; 2016 p. 44. Report No.: NG28.

[4] NICE. Diabetes in adults: quality standard. National Institute for Health and Care Excellence; 2011 p. 55. Report No.: QS6.

[5] Health and Social Care Information Centre. Recorded disease prevalence, achievements and exceptions on Quality and Outcomes Framework (QOF) for April 2014-March 2015, England. Health and Social Care Information Centre; 2015.

[6] HSCIC (2012). National Diabetes Audit 2010–2011 report 1: care processes and treatment targets.

[7] Eccles MP, Hrisos S, Francis JJ, Stamp E, Johnston M, Hawthorne G (2011). Instrument development, data collection, and characteristics of practices, staff, and measures in the improving quality of Care in Diabetes (iQuaD) study. Implement Sci.

[8] Hawthorne G, Hrisos S, Stamp E, Elovainio M, Francis JJ, Grimshaw JM (2012). Diabetes care provision in UK primary care practices. Baradaran HR, editor. PLoS One.

[9] Presseau J, Johnston M, Francis JJ, Hrisos S, Stamp E, Steen N (2014). Theory-based predictors of multiple clinician behaviors in the management of diabetes. J Behav Med.

[10] Presseau J, Johnston M, Heponiemi T, Elovainio M, Francis JJ, Eccles MP (2014). Reflective and automatic processes in health care professional behaviour: a dual process model tested across multiple behaviours. Ann Behav Med.

[11] Presseau J, Johnston M, Johnston DW, Elovainio M, Hrisos S, Steen N (2014). Environmental and individual correlates of distress: testing Karasek’s demand-control model in 99 primary care clinical environments. Br J Health Psychol.

[12] Elovainio M, Steen N, Presseau J, Francis J, Hrisos S, Hawthorne G (2013). Is organizational justice associated with clinical performance in the care for patients with diabetes in primary care? Evidence from the improving quality of care in diabetes study. Fam Pract.

[13] Tricco AC, Ivers NM, Grimshaw JM, Moher D, Turner L, Galipeau J (2012). Effectiveness of quality improvement strategies on the management of diabetes: a systematic review and meta-analysis. Lancet.

[14] Ivers NM, Sales A, Colquhoun H, Michie S, Foy R, Francis JJ (2014). No more “business as usual” with audit and feedback interventions: towards an agenda for a reinvigorated intervention. Implement Sci.

[15] Presseau J, Ivers NM, Newham JJ, Knittle K, Danko KJ, Grimshaw JM (2015). Using a behaviour change techniques taxonomy to identify active ingredients within trials of implementation interventions for diabetes care. Implement Sci.

[16] Presseau J, Sniehotta FF, Francis JJ, Campbell NC (2009). Multiple goals and time constraints: perceived impact on physicians’ performance of evidence-based behaviours. Implement Sci.

[17] Butler CC, Simpson SA, Hood K, Cohen D, Pickles T, Spanou C (2013). Training practitioners to deliver opportunistic multiple behaviour change counselling in primary care: a cluster randomised trial. BMJ.

[18] Presseau J, Hawthorne G, Sniehotta FF, Steen N, Francis JJ, Johnston M (2014). Improving Diabetes care through Examining, Advising, and prescribing (IDEA): protocol for a theory-based cluster randomised controlled trial of a multiple behaviour change intervention aimed at primary healthcare professionals. Implement Sci.

[19] Craig P, Dieppe P, Macintyre S, Michie S, Nazareth I, Petticrew M (2008). Developing and evaluating complex interventions: the new Medical Research Council guidance. BMJ.

[20] Presseau J, Francis JJ, Campbell NC, Sniehotta FF. Goal conflict, goal facilitation, and health professionals’ provision of physical activity advice in primary care: an exploratory prospective study. Implement Sci. 6:73. 10.1186/1748-5908-6-73 PMC3224555 21762486

[21] O’Brien MA, Rogers S, Jamtvedt G, Oxman AD, Odgaard-Jensen J, Kristoffersen DT, et al. Educational outreach visits: effects on professional practice and health care outcomes. In: The Cochrane Collaboration, editor. Cochrane Database of Systematic Reviews [Internet]. Chichester, UK: John Wiley & Sons, Ltd; 2007 [cited 2015 Nov 30]. Available from: http://doi.wiley.com/10.1002/14651858.CD000409.pub2 10.1002/14651858.CD000409.pub2 PMC7032679 17943742

[22] Kwasnicka D, Presseau J, White M, Sniehotta FF (2013). Does planning how to cope with anticipated barriers facilitate health-related behaviour change? A systematic review. Health Psychol Rev.

[23] Armitage CJ, Arden MA (2010). A volitional help sheet to increase physical activity in people with low socioeconomic status: a randomised exploratory trial. Psychol Health.

[24] Squires J, Presseau J, Francis J, Bond CM, Fraser C, Patey A, et al. Self-formulated conditional plans for changing health behaviour among healthcare consumers and health professionals. In: the Cochrane Collaboration, editor. Cochrane Database of Systematic Reviews [Internet]. Chichester, UK: John Wiley & Sons, Ltd; 2013 [cited 2015 Nov 13]. Available from: http://doi.wiley.com/10.1002/14651858.CD010869

[25] Joint Formulary Committee. British National Formulary (online) [Internet]. London: BMJ Group and Parmaceutical Press; [cited 2017 Jan 31]. Available from: http://www.medicinescomplete.com

[26] Office for National Statistics. Inflation and price indices [Internet]. Inflation and price indices. 2015. Available from: https://www.ons.gov.uk/economy/inflationandpriceindices

[27] Curtis L (2014). Unit costs of health and social care.

[28] Department of Health. Reference costs 2013–14 [Internet]. 2014 p. 58. Available from: https://www.gov.uk/government/publications/nhs-reference-costs-2013-to-2014

[29] Spring B, Schneider K, McFadden HG, Vaughn J, Kozak AT, Smith M, Moller AC, Epstein LH, DeMott A, Hedeker D, Siddique J (2012). Multiple behavior changes in diet and activity: a randomized controlled trial using mobile technology. Arch Intern Med.

[30] Wilson K, Senay I, Durantini M, Sánchez F, Hennessy M, Spring B (2015). When it comes to lifestyle recommendations, more is sometimes less: a meta-analysis of theoretical assumptions underlying the effectiveness of interventions promoting multiple behavior domain change. Psychol Bull.

[31] Grimshaw JM, Zwarenstein M, Tetroe JM, Godin G, Graham ID, Lemyre L (2007). Looking inside the black box: a theory-based process evaluation alongside a randomised controlled trial of printed educational materials (the Ontario printed educational message, OPEM) to improve referral and prescribing practices in primary care in Ontario, Canada. Implement Sci.

[32] Gardner B, Whittington C, McAteer J, Eccles MP, Michie S (2010). Using theory to synthesise evidence from behaviour change interventions: the example of audit and feedback. Soc Sci Med.

[33] Dombrowski SU, O’Carroll RE, Williams B (2016). Form of delivery as a key “active ingredient” in behaviour change interventions. Br J Health Psychol.

[34] O’Carroll RE (2014). Health psychology interventions. Br J Health Psychol.

[35] Marteau TM, Hollands GJ, Fletcher PC (2012). Changing human behavior to prevent disease: the importance of targeting automatic processes. Science.

[36] de Bruin M, Viechtbauer W, Hospers HJ, Schaalma HP, Kok G (2009). Standard care quality determines treatment outcomes in control groups of HAART-adherence intervention studies: implications for the interpretation and comparison of intervention effects. Health Psychol.

[37] Chen Y-F, Hemming K, Stevens AJ, Lilford RJ (2016). Secular trends and evaluation of complex interventions: the rising tide phenomenon. BMJ Qual Saf.

[38] Grimshaw JM, Presseau J, Tetroe J, Eccles MP, Francis JJ, Godin G (2014). Looking inside the black box: results of a theory-based process evaluation exploring the results of a randomized controlled trial of printed educational messages to increase primary care physicians’ diabetic retinopathy referrals [trial registration number ISRCTN72772651]. Implement Sci.

[39] Presseau J, Grimshaw JM, Tetroe JM, Eccles MP, Francis JJ, Godin G, Graham ID, Hux JE, Johnston M, Légaré F, Lemyre L (2015). A theory-based process evaluation alongside a randomised controlled trial of printed educational messages to increase primary care physicians’ prescription of thiazide diuretics for hypertension [ISRCTN72772651]. Implementation Sci.

[40] Moore GF, Audrey S, Barker M, Bond L, Bonell C, Hardeman W (2015). Process evaluation of complex interventions: Medical Research Council guidance. BMJ.

