# Supplementary material for: Cluster randomised controlled trial of a theory-based multiple behaviour change intervention aimed at healthcare professionals to improve their management of type 2 diabetes in primary care
Source: Implement Sci. 2018 May 2;13:65. doi: 10.1186/s13012-018-0754-5 (PMC5930437; doi:10.1186/s13012-018-0754-5)
Supplement: Supplementary file 1 — TIDieR-based intervention description for the IDEA trial. (DOCX 33 kb) [file 13012_2018_754_MOESM1_ESM.docx]

| Additional File 1. TIDieR-based intervention description for the IDEA trial | |
| --- | --- |
|  |  |
| Item | Description |
| Brief name | The Improving Diabetes care through Examining, Advising and prescribing (IDEA) trial |
| Why | Multiple sources of evidence of gaps in care for type 2 diabetes in the UK provided the rationale for intervening. A logic model was developed informed by previously conducted theory development work based on constructs from Social Cognitive Theory, the Health Action Process Approach, and a Dual Process Model. The logic model identifies mediators of the intervention that are modifiable, that we have previously shown explain variability in healthcare professional behaviour and that have room for improvement based on mean scores from previous research |
| What materials | Physicians, nurses and healthcare assistants were provided with access to pre-intervention reading materials available online. During the intervention session, they were provided with a workbook to use and take away, as well as a DVD with voice-over PowerPoint slides, video case studies and volitional help sheets developed with patients with type 2 diabetes to support developing coping plans planning how to circumvent personal barriers to eating healthily and engaging in physical activity. |
| What procedures | Physicians, nurses and healthcare assistants were provided access to online pre-workshop reading prior to delivery of the intervention. At the workshop, the intervention was delivered by a tandem of interventionists interactively involving PowerPoint slides, video case study examples produced for the project allowing the identification of barriers and informing a discussion about forming solutions to the barriers within the practice, as well as providing a video demonstration of how to circumvent challenges. The session was explicitly designed to operationalize the delivery of 12 behaviour change techniques (see Table 2). Attendance certificates were provided to all those attending the intervention session, signed by the PI. |
| Who provided | Every session was delivered by two interventionists: one with a medical (MD) or nursing (RN) background and one with a health psychology (PhD) background. The two interventionists were selected from a pool of 4 MD/RNs and 3 PhD health psychologists, with selection based on timing and availability coinciding with practice availability. The trial RA was also on hand at each session to manage practical arrangements. |
| How | The pre-session reading was delivered online. The main intervention components were delivered in a face to face group setting with members of an individual practice, using multiple modes of delivery including presentation, discussion, group work, individual work (using workbooks), and observing case study videos and demonstration of successful performance. |
| Where | The intervention was delivered at 22 individual practice locations, in a dedicated meeting room at each site. |
| When and how much | The intervention was delivered once to each practice and lasted approximately 90 minutes. The main time emphasis was on demonstration of the behaviour for prescribing forHbA1c and providing physical activity advice (videos) and problem solving for all six target behaviours. |
| Tailoring | The behaviour change technique "problem solving" (i.e. coping planning) is designed to be tailored to the barriers and solutions formed by individual health professionals and thus this strategy was by design tailored to their own setting and circumstances. |
| Modifications | In one of the 22 sessions, the video demonstrations of successful performance did not work; instead, the two interventionists acted out the content of the video in person. |
| How well (planned) | We conducted a fidelity of delivery and receipt study, involving audio recording and transcribing each session, and double coding (with time stamps) the presence of designed and unplanned behaviour change techniques delivered by the interventionists and the duration of delivery of each technique in each session by each interventionist. We also conducted a mechanistic process evaluation to investigate whether the intervention led to change in any targeted process variables |
| How well (actual) | Details of the fidelity of the intervention are reported in our companion papers. |
