# Supplementary material for: Cluster randomised controlled trial of a theory-based multiple behaviour change intervention aimed at healthcare professionals to improve their management of type 2 diabetes in primary care
Source: Implement Sci. 2018 May 2;13:65. doi: 10.1186/s13012-018-0754-5 (PMC5930437; doi:10.1186/s13012-018-0754-5)
Supplement: Supplementary file 2 — Labels and descriptions of behaviour change techniques delivered during the IDEA intervention. (DOCX 30 kb) [file 13012_2018_754_MOESM2_ESM.docx]

| Additional File 2. Labels and descriptions of behaviour change techniques delivered during the IDEA intervention | |
| --- | --- |
|  |  |
| **Behaviour change technique (target constructs)** | **Description** |
| Demonstration of the behaviour (self-efficacy) | Observable example of the successful performance of two behaviours (prescribing for HbA1c and providing physical activity advice) despite the presence of barriers. This is to provide a credible model that it is possible to address a challenging situation |
| Verbal persuasion of capability (self-efficacy) | Throughout session, tell the clinicians that they can successfully perform the key clinical actions, making sure to argue against any comments about doubts in their ability to manage difficult situations by asserting that they can and supporting them through problem solving |
| Graded tasks (self-efficacy) | Suggest that clinicians set easy-to-perform tasks first and build their way up to more challenging but achievable tasks until they enact the clinical actions in their identified challenging situation |
| Behavioural practice/rehearsal (self-efficacy) | Prompt clinicians to practice plans with colleagues and familiar patients first |
| Problem solving (automaticity; coping planning) | Prompt clinicians to identify barriers to the six key clinical actions, and prompt them to identify **NEW** solutions to those barriers, which allow them to enact the clinical actions (emphasize group discussion, provide examples for each behaviour as needed). Prompt each participant to then write their own 'if-then' coping plans. |
| Adding objects to the environment (coping planning) | Volitional help sheets that clinicians can use to support patients in forming 'if-then' plans |
| Goal setting behaviour (intention) | Prompt discussion of key diabetes management behaviours they are involved in, then show the six key clinical actions linked to NICE quality standards. Group discussion and self-identification of which goals they are currently pursuing/responsible for |
| Discrepancy between behaviour and goal (intention) | Draw attention to the gap between their intention and their clinical actions |
| Incentive outcome (intention) | Draw attention to target behaviours linked to QOF points |
| Habit formation (automaticity, self-efficacy) | Suggested to clinicians that they repeat and practise the clinical actions repeatedly so that it becomes second nature and embedded into their routines |
| Instruction on how to perform the behaviour (outcome expectations) | Provide test of knowledge of what current NICE Quality Standards and NICE guidelines recommend for each of the six behaviours |
| Credible source (outcome expectations) | Introductions emphasize clinical and behaviour change credibility of interventionists |
| *Note.* Behaviour change techniques and definitions from Michie et al. 2013 | |
