# Supplementary material for: Cluster randomised controlled trial of a theory-based multiple behaviour change intervention aimed at healthcare professionals to improve their management of type 2 diabetes in primary care
Source: Implement Sci. 2018 May 2;13:65. doi: 10.1186/s13012-018-0754-5 (PMC5930437; doi:10.1186/s13012-018-0754-5)
Supplement: Supplementary file 3 — TIDieR-based description of training provided to facilitators in the IDEA trial. (DOCX 30 kb) [file 13012_2018_754_MOESM3_ESM.docx]

| Additional File 3: TIDieR-based description of training provided to facilitators in the IDEA trial | |
| --- | --- |
|  |  |
| Item | Description |
| Brief name | The Improving Diabetes care through Examining, Advising and prescribing (IDEA) trial |
| Why | To maximise the fidelity and consistency of delivery of the intervention by the interventionists |
| What materials | *Interventionists* were provided with an interventionist handbook describing the rationale and how to deliver each behaviour change technique designed to be delivered. They were provided with annotated PowerPoint based slides of the intervention materials indicating turn order for each interventionist. They were also provided with all materials provided to recipient healthcare professionals, including videos and participant workbooks used in delivering the intervention. They were also provided with a post-delivery debrief sheet to record their experience of delivering each intervention session. |
| What procedures | Three separate training opportunities were provided to interventionists. First, a group-based observation of the intervention delivery being demonstrated, delivered by the PI and including opportunities to ask for clarification, identification of any anticipated barriers to delivery (practical or skills based). Second, Individual opportunity for each interventionist to practise delivering their component of the intervention within a research environment where participants were researchers role playing physicians, nurses and healthcare assistants. Corrective feedback on performance and clarifications were provided by the PI. Third, midway through delivery, a group based discussion of progress and problem solving any barriers, as well as provision of summary feedback about the sessions from participating healthcare professionals |
| Who provided | The PI (psychologist and implementation scientist) and trial RA (health services researcher) led the interventionist training |
| How | All training was provided in face to face sessions, either as a group involving all 7 interventionists or else involving each interventionist one at a time |
| Where | Training of interventionists was provided in meeting rooms which included PowerPoint facilities, at the Institute of Health and Society at Newcastle University and at the Centre for Life in Newcastle, UK |
| When and how much | One half-day group session, one two hour practice session for each interventionist, and one 2-hour group-based progress and feedback session |
| Tailoring | The group sessions were not planned to be tailored. The individual practice sessions were tailored to focus on the sections of intervention that each interventionist was responsible for delivering. |
| Modifications | Practice sessions were planned immediately following the initial group-based observation but due to time constraints, the practice sessions were conducted at a later time and different location |
| How well (planned) | We did not assess the fidelity of the training of the interventionists. |
| How well (actual) | We did not assess the fidelity of the training of the interventionists. |
