# Supplementary material for: Cluster randomised controlled trial of a theory-based multiple behaviour change intervention aimed at healthcare professionals to improve their management of type 2 diabetes in primary care
Source: Implement Sci. 2018 May 2;13:65. doi: 10.1186/s13012-018-0754-5 (PMC5930437; doi:10.1186/s13012-018-0754-5)
Supplement: Supplementary file 4 — Main outcome definitions and operationalizations. (DOCX 32 kb) [file 13012_2018_754_MOESM4_ESM.docx]

| Additional File 4. Main outcome definitions and operationalizations | | |
| --- | --- | --- |
|  |  |  |
| Outcome description | Source of outcome | Measure of outcome |
| Prescribing additional therapy for the management of glycemic control (HbA1c) in patients with type 2 diabetes who HbA1c is higher than 58 mmol/mol (7.5%) despite maximum dosage on all oral hypoglycamic drugs. | Electronic medical records (all patients, for period covering 12 months before and 12 months after intervention) | *Numerator:* number of patients prescribed insulin for the first time. *Denominator:* number of patients with type 2 diabetes in each practice with an HbA1c measure exceeding 58 mmol/mol (7.5%) at least once in the 12 months post-intervention who were also on three or more oral hypoglycemic drugs |
| Prescribing additional antihypertensive drugs (i.e. new drug or increased dosage) for patients with type 2 diabetes with uncomplicated hypertension whose blood pressure is at least 5mmHg above 140/80 mmHg even following previous management | Electronic medical records (all patients, for period covering 6 months before and 12 months after intervention) | *Numerator:* number of patients prescribed a new BP drug, an additional drug, or a change in existing tablet size. *Denominator:* number of patients with type 2 diabetes in each practice with at least one BP measure above 145mmHg systolic or 85mmHg diastolic who do not then have a follow-up BP measure below 145/85mmHg |
| Personally ensuring that circulation and sensation in the feet of people with type 2 diabetes have been examined in the past 12 months, by examining feet yourself and/or referring them. | Electronic medical records (all patients, for period covering 12 months before and 12 months after intervention) | *Numerator:* patients with recorded examination of sensation and circulation in each foot at least once in last 12 months. *Denominator:* all patients meeting inclusion criteria. |
| Provide personalised nutrition advice to patients with type 2 diabetes whose BMI is above a target of 30 kg/m2 even following previous management. | Patient reported questionnaire (random sample) | Patients with BMI >30 responding 'Yes' or 'No/unsure' to "In the past 12months at your diabetes appointment, did you have a discussion about your diet/nutrition?" |
| Provide on-going education about diabetes for patients with type 2 diabetes | Patient reported questionnaire (random sample) | Patients responding 'Yes' or 'No/unsure' to "In the past 12 at your diabetes appointment, did you have a discussion about your physical activity?" |
| Provide personalised advice on physical activity for patients with type 2 diabetes | Patient reported questionnaire (random sample) | Patients responded 'Yes' or 'No/unsure' to "In the past 12 months at your diabetes appointment, were you given any new or additional information about diabetes or reminded about important information?" |
