# Supplementary material for: Cluster randomised controlled trial of a theory-based multiple behaviour change intervention aimed at healthcare professionals to improve their management of type 2 diabetes in primary care
Source: Implement Sci. 2018 May 2;13:65. doi: 10.1186/s13012-018-0754-5 (PMC5930437; doi:10.1186/s13012-018-0754-5)
Supplement: Supplementary file 5 — Cost of delivering the IDEA intervention in primary care (n = 22). (DOCX 34 kb) [file 13012_2018_754_MOESM5_ESM.docx]

| Additional File 5: Cost of delivering the IDEA intervention in primary care (n=22) | |  |  |  |  |  |
| --- | --- | --- | --- | --- | --- | --- |
|  |  |  |  |  |  |  |
| **Activity** | **Cost per practice (£)** |  |  |  |  |  |
| **Preparation and delivery total** | 1191 |  |  |  |  |  |
| Training interventionists | 73 |  |  |  |  |  |
| Materials production | 172 |  |  |  |  |  |
| Workshop content production | 314 |  |  |  |  |  |
| Participant time | 533 |  |  |  |  |  |
| Refreshments | 100 |  |  |  |  |  |
|  |  |  |  |  |  |  |
|  | **12 months pre-intervention** | |  | **12-months post-intervention** | |  |
| **Prescription costs (mean cost per patient in £)** | **Intervention (20)** | **Control (20)** | ***p*** | **Intervention (20)** | **Control (20)** | ***p*** |
| Prescription costs for all diabetes medication | 1484 | 1727 | <.01 | 1437 | 1611 | <.01 |
| Prescription costs for injectable diabetes medication | 6531 | 7205 | <.01 | 6081 | 6570 | 0.25 |
| Prescription costs for blood pressure medication | 96 | 89 | 0.33 | 92 | 83 | 0.30 |
| **Patient costs (per patient)** |  |  |  |  |  |  |
| Total costs | -- | -- | -- | 1570 | 1676 | <.01 |
| Equivalized diabetes costs | -- | -- | -- | 1447 | 1577 | <.01 |
| Equivalized blood pressure costs | -- | -- | -- | 99 | 77 | <.01 |
| Service use costs | -- | -- | -- | 24 | 22 | <.01 |
